# Supplementary material for: Relationship between the average slope in the active commuting to and from school and fitness in adolescents: the mediator role of fatness
Source: PeerJ. 2020 Jun 9;8:e8824. doi: 10.7717/peerj.8824 (PMC7292016; doi:10.7717/peerj.8824)
Supplement: Supplemental Information 2 [file peerj-08-8824-s002.pdf]

A continuación te exponemos una serie de preguntas relacionadas con tus hábitos de actividad física. No existen respuestas correctas, por lo que te pedimos que seas lo más sincero posible, ya que los datos serán tratados de forma anónima. Muchas gracias por tu colaboración

Código alumno/a ______

Centro/Instituto ______ **Curso** ______

Género: ☐ Masculino ☐ Femenino **Edad (años):**

Localidad ______ **Provincia** ☐ Cáceres ☐ Badajoz

Nombre de la calle y nº ______

1. ¿Cómo vas al instituto? ☐ A pie ☐ En coche ☐ Bicicleta ☐ Moto/Bus

2. ¿Cuánto tiempo tardas en ir al instituto?
☐ Menos de 5 min ☐ Entre 5 y 10 min ☐ Entre 11-20 min ☐ Entre 21-30 min ☐ Más de 30 min

3. ¿Cómo vuelves del instituto? ☐ A pie ☐ En coche ☐ Bicicleta ☐ Moto/Bus

4. ¿Cuánto tiempo tardas en volver del instituto?
☐ Menos de 5 min ☐ Entre 5 y 10 min ☐ Entre 11-20 min ☐ Entre 21-30 min ☐ Más de 30 min

5. En tu opinión, ¿el centro educativo favorece el ir caminando o usar la bicicleta para ir y volver?
☐ Mucho ☐ Bastante ☐ Normal ☐ Poco ☐ Nada

6. ¿Te resulta divertido caminar o usar la bicicleta para ir y volver del instituto?
☐ Mucho ☐ Bastante ☐ Normal ☐ Poco ☐ Nada

7. ¿Te parece saludable caminar o usar la bicicleta para ir y volver del instituto?
☐ Mucho ☐ Bastante ☐ Normal ☐ Poco ☐ Nada

8. A lo largo de la semana, ¿te sueles ir tarde a la cama por estar viendo la televisión, jugando a la consola/ordenador o navegando en internet?
☐ Mucho ☐ Bastante ☐ Normal ☐ Poco ☐ Nada

9. A lo largo de la semana, ¿te encuentras cansado cuando vas al instituto?
☐ Mucho ☐ Bastante ☐ Normal ☐ Poco ☐ Nada

10. A lo largo de la semana, ¿permaneces cansado a lo largo del día?
☐ Mucho ☐ Bastante ☐ Normal ☐ Poco ☐ Nada

11. En la última semana, ¿cuánto tiempo has pasado en casa por la tarde?

**LUNES**	**MARTES**	**MIÉRCOLES**	**JUEVES**	**VIERNES**
☐ Toda la tarde	☐ Toda la tarde	☐ Toda la tarde	☐ Toda la tarde	☐ Toda la tarde
☐ Casi toda la tarde	☐ Casi toda la tarde	☐ Casi toda la tarde	☐ Casi toda la tarde	☐ Casi toda la tarde
☐ La mitad de la tarde	☐ La mitad de la tarde	☐ La mitad de la tarde	☐ La mitad de la tarde	☐ La mitad de la tarde
☐ Casi nada	☐ Casi nada	☐ Casi nada	☐ Casi nada	☐ Casi nada
☐ Nada	☐ Nada	☐ Nada	☐ Nada	☐ Nada

12. En el último fin de semana, ¿cuánto tiempo has pasado en casa por la tarde?

**SÁBADO**	**DOMINGO**
☐ Toda la tarde	☐ Toda la tarde
☐ Casi toda la tarde	☐ Casi toda la tarde
☐ La mitad de la tarde	☐ La mitad de la tarde
☐ Casi nada	☐ Casi nada
☐ Nada	☐ Nada

Actividad física es cualquier actividad que incrementa tu ritmo cardiaco y acelera tu respiración. Puede ser hacer deporte, jugar con amigos, caminar al colegio. E**jemplos**: correr, caminar, montar en patines, bailar, nadar, fútbol, baloncesto, voleibol, balonmano

13. En una semana normal, ¿cuántos días haces actividad física 60 minutos o más?
☐ 0 días ☐ 1 – 2 días ☐ 3 – 4 días ☐ 4 – 5 días ☐ 6 – 7 días

14. De los últimos 7, ¿cuántos hiciste actividad física 60 minutos o más?
☐ 0 días ☐ 1 – 2 días ☐ 3 – 4 días ☐ 4 – 5 días ☐ 6 – 7 días

15. De los últimos 7, ¿cuántos días hiciste ejercicio para fortalecer tus músculos?
☐ 0 días ☐ 1 – 2 días ☐ 3 – 4 días ☐ 4 – 5 días ☐ 6 – 7 días

16. De los últimos 7, ¿cuántos hiciste estiramiento para alargar o relajar tus músculos?
☐ 0 días ☐ 1 – 2 días ☐ 3 – 4 días ☐ 4 – 5 días ☐ 6 – 7 días

Ya HAS FINALIZADO el cuestionario. De la siguiente parte, **NO TIENES** que **RELLENAR NADA** de la información que se muestra a continuación:

COMPOSICIÓN CORPORAL Y CONDICIÓN FÍSICA							
**Peso (Kg)**				**Altura (cm)**			
PERÍMETROS CORPORALES							
**Cintura (cm)**				**Cadera (cm)**			
**Cuello (cm)**				**ICC**			
PLIEGUES CUTÁNEOS							
**Tricipital (mm)**				**Subescapular (mm)**			
COURSE NAVETTE Y SALTO HORIZONTAL							
**Nº Ciclos**				**Salto horizontal**			

JUNTA DE EXTREMADURA
Consejería de Economía e Infraestructuras

Unión Europea

CONSEJERÍA DE ECONOMÍA E INFRAESTRUCTURAS

PROYECTOS DE INVESTIGACIÓN EN CENTROS PÚBLICOS DE I+D+i
